# Predictive Value of Various Atypical Cells for the Detection of Human Papillomavirus in Cervical Smears

**DOI:** 10.3390/ijms25021212

**Published:** 2024-01-19

**Authors:** Kaori Okayama, Mao Kakinuma, Koji Teruya, Mizue Oda, Masahiko Fujii, Hirokazu Kimura, Toshiyuki Sasagawa, Mitsuaki Okodo

**Affiliations:** 1 Department of Health Science, Gunma Paz University Graduate School of Health Sciences, 1-7-1 Tonyamachi, Takasaki-shi 370-0006, Gunma, Japan; 2 Department of Health and Welfare, Faculty of Health Sciences, Kyorin University, 5-4-1 Shimorenjaku, Mitaka-shi 181-8621, Tokyo, Japan; 3 Genki Plaza Medical Center for Health Care, 3-6-5 Iidabashi, Chiyoda-ku 102-0072, Tokyo, Japan; 4 Department of Medical Technology, Faculty of Health Sciences, Kyorin University, 5-4-1 Shimorenjaku, Mitaka-shi 181-8621, Tokyo, Japan; 5 Department of Obstetrics and Gynecology, Kanazawa Medical University, 1-1 Uchinadadaigaku, Kahoku-gun 920-0293, Ishikawa, Japan

**Keywords:** human papillomavirus, binucleated cell, parakeratotic cell, Papanicolaou test, microdissection, cervical intraepithelial neoplasia

## Abstract

It is thought that numerous genotypes of human papillomavirus (HPV) are associated with various atypical cells, such as multinucleated cells, koilocytes, binucleated cells, parakeratotic cells, and giant cells, in the cervix. We previously showed the specificity of HPV genotypes for koilocytes and multinucleated cells. Therefore, in this study, we analyzed the association among HPV genotypes and binucleated cells, parakeratotic cells, and giant cells in Papanicolaou (Pap) smears. We detected HPV genotypes and atypical cells in 651 cases of liquid-based cytology with an abnormal Pap smear. The HPV genotypes associated with atypical cells were evaluated using stepwise logistic regression with backward elimination and a likelihood ratio test for model construction. Polymerase chain reaction was used to determine the HPV genotypes in whole liquid-based cytology samples and microdissected cell samples from Pap smear slides. Binucleated cells were significantly associated with HPV genotype 42. Moreover, parakeratotic cells were significantly associated with certain HPV genotypes, such as HPV40. However, it was difficult to detect specific HPV genotypes by the manual microdissection-polymerase chain reaction method despite the presence of binucleated cells and parakeratotic cells. Thus, the presence of binucleated cells, parakeratotic cells, and giant cells in Pap smears may not be predictive of cervical lesions above low-grade squamous intraepithelial lesions or infection with highly carcinogenic HPV genotypes.

## 1. Introduction

Over 200 genotypes of human papillomavirus (HPV) have been identified. These genotypes can lead to various conditions, such as common warts on hands and feet and genital warts, and they have the potential to cause cancer [1,2,3,4]. Approximately 20 HPV genotypes are more commonly found in women with cervical cancer compared to those with normal cytology [5]. Persistent infection with a high-risk HPV genotype is a leading cause of high-grade cervical intraepithelial neoplasia (CIN) and invasive cervical cancer (ICC) [6]. The primary aim of cervical cancer screening is to reduce the risk of ICC and associated mortality rate by detecting CIN grade 2 (CIN2) or worse and providing an opportunity for appropriate treatment. Screening tools for cervical cancer include Papanicolaou (Pap) and HPV tests. The Pap test has historically been the method of choice in cervical cancer screening; however, the latest scientific evidence underscores the superiority of HPV testing in detecting cervical cancer caused by HPV. This growing body of evidence has led to a shift in routine practice in many countries as health authorities are increasingly adopting primary high-risk HPV screening over the traditional Pap screening [7,8,9,10]. The key advantage of HPV testing lies in its higher sensitivity for detecting CIN and its direct association with HPV, the primary cause of cervical cancer. Notably, this shift from the traditional Pap screening to HPV testing may result in more women testing positive for HPV and subsequently being referred for further evaluation. This approach aims to strike a balance between identifying women who are at risk of progression to the CIN3 (pre-cancer) while minimizing unnecessary referrals and the associated anxiety, given that most HPV infections clear without treatment [11]. However, HPV testing leads to overdiagnosis, because it cannot distinguish between transient and persistent infections. Additionally, HPV tests have higher sensitivity but lower specificity than cytology for the detection of CIN grade 3 or worse [12]. Therefore, improving the specificity of triage cytology following HPV testing for the detection of CIN is necessary to address these limitations.

Koilocytosis, multinucleated cells, binucleated cells, parakeratosis, and giant cells are known cytological changes associated with HPV infection. Bollman et al. reported that the critical evaluation of mild cellular changes suggestive of HPV infection may increase the sensitivity of cytological diagnosis. This approach offers the potential to preselect cases prior to conducting HPV molecular testing [13]. However, the paramount goal in triage cytology is to distinguish between women who may have transient HPV infection that is likely to clear on its own and those who have persistent infection and are at risk for precancerous or cancerous cervical lesions. To achieve this goal, it is crucial to prioritize specificity, which ensures that only those truly at risk are referred for colposcopy, thereby reducing the overall positivity rate and burden on healthcare resources. Therefore, further analysis of cytological evidence of HPV infection is warranted.

Using microdissection (MD) and HPV genotyping with sensitive polymerase chain reaction (PCR), we found evidence that certain HPV genotypes are not associated with koilocytic changes [14,15]. It appears that major high-risk HPV types, such as HPV16, HPV18, and HPV52, do not induce koilocytic changes [15]. Moreover, we showed that multinucleated cells are associated with HPV16, HPV34, and HPV56 [16]. Pap smear cytology is of limited value due to the possibility of false-negative results. Nevertheless, it may provide clinicians with useful new information, such as predicting the risk of developing high-grade lesions. Certain cytological features may indicate infection with a particular genotype, especially in triage cytology following HPV testing without genotyping capability. This study aimed to analyze the association between HPV genotypes and cytologic signs of HPV infection, such as binucleated and dyskeratotic cells in the Pap smear, in order to elucidate the contribution of cytological evaluation to more effective risk assessment and management in cervical cancer screening.

## 2. Results

### 2.1. Relationships among Cytological Signs of HPV Infection, Cytological Classification, and HPV Genotypes

The relationships between the cytologic classification and atypical cells are summarized in Table 1. Binucleated cells, parakeratosis, and giant cells were present in 84.6% (551/651 cases), 35.5% (231/651 cases), and 41.5% (270/651 cases) of patients, respectively. Binucleated cells were the most commonly observed cells in all cytology classifications. Binucleated cells and giant cells were most frequently present in Pap smears from atypical squamous cells of undetermined significance (ASC-US) cases (82.5% (113/137 cases) and 53.3% (73/137 cases), respectively). Parakeratotic cells were most frequently present in Pap smears from low-grade squamous intraepithelial lesion (LSIL) cases (50.0% (101/202 cases)). All cytological signs appeared more often in LSIL versus high-grade squamous intraepithelial lesion (HSIL).

Correlations between cytological signs of HPV infection (i.e., binucleated, parakeratotic, and giant cells) and HPV genotypes are shown in Table 2 and Table 3. Binucleated cells were frequently associated with all HPV genotypes (positivity rate: >70%). The positivity rate for the association of binucleated cells with high-risk and other HPV genotypes was >90% (i.e., HPV45 (100.0%), HPV42 (93.1%), and HPV89 (94.1%), respectively). However, only six cases exhibited positivity for HPV45. The positivity rate for the association of parakeratotic cells with high-risk and other HPV genotypes was >50% (i.e., HPV56 (56.8%), HPV40 (71.4%), HPV42 (55.2%), HPV62 (54.5%), HPV73 (55.6%), and HPV90 (56.8%), respectively). Moreover, the positivity rate for the association of giant cells with high-risk and other HPV genotypes was 60.9% and 80.0% for HPV33 and HPV35, respectively.

### 2.2. Relationships between Detected HPV Genotypes and Cytological Signs of HPV

Binomial logistic regression analysis was used to determine the relationships between detected HPV genotypes and atypical cells (Table 4). The model was a good fit (Hosmer–Lemeshow test; *p* > 0.05) for all cytological signs. Binucleated cells were significantly associated with the presence of HPV52 (*p* = 0.002; odds ratio (OR): 2.01; 95% confidence interval (95%CI): 1.28–3.15) and HPV42 (*p* = 0.017; OR: 2.69; 95%CI: 1.19–6.07). In contrast, there was no association between HPV11 and binucleated cells. Moreover, parakeratotic cells were significantly associated with the presence of HPV16, HPV56, HPV58, HPV40, HPV42, and HPV62; among these, HPV56 (*p* = 0.000; OR: 3.26; 95%CI: 1.92–5.53) and HPV40 (*p* = 0.011; OR: 5.19; 95%CI: 1.45–18.59) exhibited an OR ≥ 3. Conversely, giant cells were not significantly associated with any genotype.

### 2.3. HPV Genotype Detection by Manual MD (MD-PCR) in Binucleated and Parakeratotic Cells for Multiple Infections

**Figure 1:**
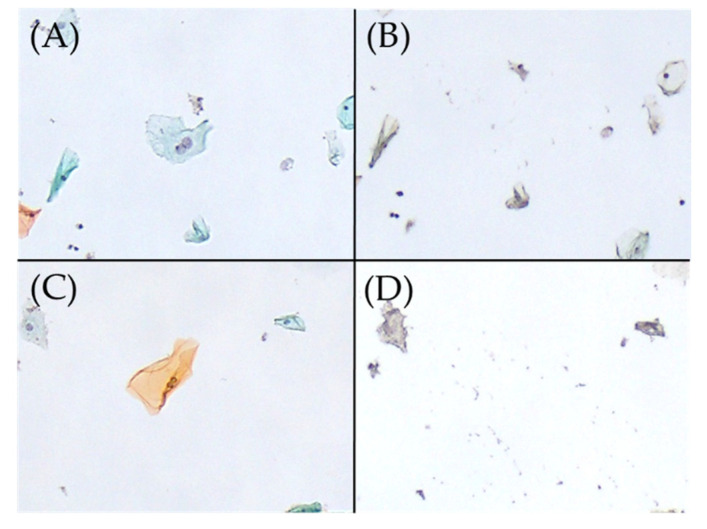
Excision of binucleated cells on Papanicolaou smear slides, performed using the microdissection-based technique. (**A**) Binucleated cell (Papanicolaou stain, ×100). (**B**) Appearance after the manual microdissection of one binucleated cell shown in A with the tip of a 27 G needle under a microscope (×100). HPV51 was detected in this manual microdissected sample using the uniplex E6/E7 PCR method. (**C**) Binucleated cell (Papanicolaou stain, ×100). (**D**) Appearance after the manual microdissection of one binucleated cell shown in C with the tip of a 27 G needle under a microscope (×100). HPV was not detected in this sample using the uniplex E6/E7 PCR method. HPV, human papillomavirus; PCR, polymerase chain reaction.

**Figure 2:**
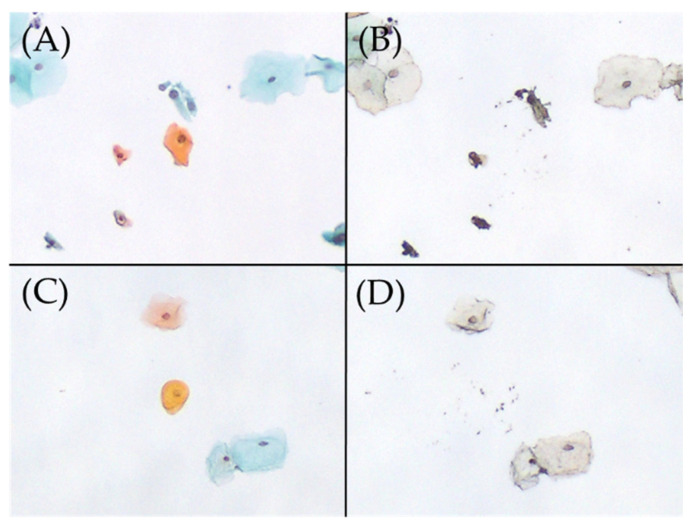
Excision of parakeratotic cells on Papanicolaou smear slides, performed using the microdissection-based technique. (**A**) Parakeratotic cell (Papanicolaou stain, ×100). (**B**) Appearance after the manual microdissection of one parakeratotic cell shown in A with the tip of a 27 G needle under a microscope (×100). HPV51 was detected in this manual microdissected sample using the uniplex E6/E7 PCR method. (**C**) Parakeratotic cell (Papanicolaou stain, ×100). (**D**) Appearance after the manual microdissection of one parakeratotic cell shown in C with the tip of a 27 G needle under a microscope (×100). HPV was not detected in this sample using the uniplex E6/E7 PCR method. HPV, human papillomavirus; PCR, polymerase chain reaction.

We performed MD for the detection of HPV genotypes that showed high specificity for binucleated and parakeratotic cells based on the binomial logistic regression analysis (Figure 1 and Figure 2). Table 5 provides a comparison of the HPV genotypes associated with binucleated cells (determined by MD-PCR) with whole liquid-based cytology-PCR (LBC-PCR) results from nine cases of infection with multiple genotypes, including HPV52 and HPV42 (Table 4); these genotypes have been linked to the induction of binucleated cells. In addition, only three cases with a co-presence of HPV11 and binucleated cells were detected (Table 3), and the lack of cell remnants prevented analysis using the MD method. From nine cases, we obtained 57 MD samples of binucleated cells. The samples included HPV52 (32 cells) and HPV42 (27 cells). Of those, six samples (10.5%) were single HPV-positive (i.e., detection of HPV51 or HPV6), and the remaining 51 samples were HPV-negative.

Table 6 provides a comparison of the HPV genotypes associated with parakeratotic cells (determined by MD-PCR) with whole LBC-PCR results from 20 cases of detection of multiple genotypes, including HPV16, HPV56, HPV58, HPV40, HPV42, and HPV62 (Table 4); these genotypes have been linked to the induction of parakeratotic cells. From 20 cases, we obtained 247 MD samples of parakeratotic cells. The samples included HPV16 (110 cells), HPV56 (36 cells), HPV58 (97 cells), HPV40 (48 cells), HPV42 (119 cells), and HPV62 (21 cells). Of those, 19 samples (7.8%) were single HPV-positive (i.e., detection of HPV16, HPV56, HPV42, HPV52, or HPV74). Among the HPV genotypes that exhibited significant differences in the binomial logistic regression analysis, HPV16, HPV56, and HPV42 were positive based on MD-PCR. However, their positivity rates were <10%. In addition, differentiation (e.g., superficial or parabasal type) did not affect the rates of HPV detection.

Most HPV genotypes associated with binucleated cells and parakeratotic cells detected using MD differed from those detected by binomial logistic regression analysis. Additionally, in the present analysis, HPV was also detected in MD samples without nuclear atypia. Hence, there may be no relationship between mild nuclear atypia and HPV positivity (Figure 1 and Figure 2).

## 3. Discussion

In this study, we investigated potential relationships between various atypical cells, cytopathological classification based on the Bethesda system, and HPV infection profiles in cervical Pap smears using MD and uniplex E6/E7 PCR. The main results are discussed below. First, the rate of each type of atypical cell on each cytopathological classification fluctuated, with binuclear cells demonstrating the highest detection rate in all pathological classifications (Table 1). Second, relationships between specific HPV genotypes and cell findings (binucleated and parakeratotic cells) were detected in whole liquid-based cytology. For example, HPV52, HPV11, and HPV42 were detected in binucleated cells (Table 4). Third, the HPV detection rate for binucleated cells and parakeratotic cells, according to MD-PCR, was approximately 10%, and other HPV genotypes besides these showed statistical significance in liquid-based cytology (Table 5 and Table 6). These results suggested that binucleated, parakeratotic, and giant cells detected in Pap smears were not associated with specific HPV genotypes. Additionally, cytology revealed that the rates of binucleated cells, dyskeratotic cells, and giant cells were similar across the ASC-US, LSIL, ASC-H, and HSIL categories (Table 1), suggesting that these features were not discriminatory in predicting CIN2+ lesions.

Binucleated cells were most frequently present in the Pap smears of patients with HPV infection. Binucleation is one of several morphological epithelial changes induced by sexually transmitted pathogens, such as Candida and HPV [17,18,19]. These changes, defined as reactive cellular changes under the “negative for intraepithelial lesion or malignancy” category in the Bethesda system, have not yet been distinguished from HPV infection [18]. In this study, a high positivity rate (~85%) was detected in all cases; the positivity rate according to MD-PCR was approximately 10%. Thus, the detection of binuclear cells alone is insufficient to indicate HPV infection. Giant cells have been associated with binucleation and multinucleation. It has been reported that these cells are present in 13.7% of cases of HPV infection [20]. In this study, we did not observe a significant association of giant cells with any HPV genotypes.

Previous studies have provided insight into the relationship between parakeratosis and HPV infection. In a recent study of follow-up biopsy specimens, a trend was observed between parakeratosis and increased frequency of HSIL [21]. In addition, HPV positivity is more common in cases with parakeratosis than in those without parakeratosis. Thus, Kir et al. suggested that the presence of parakeratosis should be considered in standard cytology reports [22]. Zahn et al. reported that the presence of hyperkeratosis/parakeratosis on an otherwise normal Pap smear is associated with low-grade changes, particularly among women of reproductive age [23]. These studies suggest that among HPV-infected cells, parakeratosis is associated with intraepithelial lesions. In the present study, parakeratosis was most frequently observed in Pap smears from LSIL cases and exhibited a lower incidence in HSIL cases. Although parakeratosis was associated with a wide range of genotypes, including low-risk types, the HPV positivity rate based on MD-PCR was only approximately 10%. Previous studies and the present investigation indicate that parakeratosis may be an HPV-induced finding, as it is more frequently detected in cases with HPV infection. However, the results of MD-PCR suggested that dyskeratosis without nuclear atypia is associated with HPV infection below the detection limit (i.e., 100 copies) of the uniplex E6/E7 PCR method or with an absence of HPV infection.

Our study has certain limitations. We did not investigate whether specific HPV genotypes were detected in microdissected HPV-infected cells (binucleated, parakeratotic, and giant cells) from histological specimens. Thus, it is unclear whether HPV-infected cells are present in CIN tissues. However, when HPV is isolated from a single cell using the laser capture microdissection, contamination of adjacent cells is inevitable [24]. Through MD and HPV genotyping with sensitive PCR, we previously demonstrated that koilocytes and multinucleated cells are cytological signs of particular HPV genotypes [14,16]. To the best of our knowledge, this is the first comprehensive analysis of HPV genotypes for single cells in HPV-infected cells. We think that further meaningful results could be obtained by analyzing the relationship between HPV genotypes and cytological signs of HPV infection in Pap smears. In addition, we excluded nuclear atypia to provide an overview of the cytological signs of HPV infection. Thus, nuclear atypia of binucleated cells and parakeratotic cells should be analyzed in future studies to enhance our understanding of HPV-infected cells.

The present study revealed that binucleated, parakeratotic, and giant cells were significantly associated with specific HPV genotypes. Nevertheless, it was difficult to detect specific HPV genotypes by MD-PCR. The detection rate of HPV by MD-PCR was approximately 10%. Based on this evidence, binucleated, parakeratotic, and giant cells may not be associated with specific HPV genotypes. In the triage of women infected with a high-risk HPV genotype, the presence of binucleated, parakeratotic, and giant cells in Pap smears may not be predictive of cervical lesions above LSIL or infection with highly carcinogenic HPV genotypes. Thus, cytologists should consider this following the detection of such cells in cytological examination to avoid overdiagnosis. Therefore, we believe that the findings of this study will contribute to the improvement of the specificity of triage cytology following HPV testing for the detection of CIN.

## 4. Materials and Methods

### 4.1. Clinical Samples

All samples were obtained during the follow-up of 1053 patients at the Genki Plaza Medical Center for Health Care, Tokyo, Japan, between 2014 and 2018. The mean patient age was 38 years (range: 20–67 years). Data on HPV vaccination history were not collected. Of the 1053 samples, we used 651 SurePath™ (Becton Dickinson and Company, Franklin Lakes, NJ, USA) LBC samples having ASCUS+, according to the Bethesda system [18]. The 651 Pap smears included the following diagnoses: ASCUS (*n* = 137), LSIL (*n* = 202), and atypical squamous cells and high-grade squamous intraepithelial lesion could not be ruled out (*n* = ASCH 54), and HSIL (*n* = 258) [14,16]. All Pap smears were strictly judged in a blinded manner by two cytotechnologists for the presence or absence of the following three cytological signs: binucleated cells; parakeratotic cells; and giant cells. MD samples showing nuclear atypia similar to squamous intraepithelial lesion were excluded from the detection of binucleated and parakeratotic cells. For confirmation of diagnosis, the cytotechnologists resolved any discrepancies by simultaneously viewing the cells.

### 4.2. Ethical Approval

Patients provided written informed consent prior to the collection of samples. The Ethics Committee on Human Research of Kyorin University and Gunma Paz University approved the study protocol, which was implemented in accordance with approved guidelines (approval numbers: 2023-1 and PAZ22-41).

### 4.3. HPV Genotyping with Whole LBC Samples

We performed HPV genotyping with whole LBC samples to assess the association between cytological signs on Pap smears and HPV genotypes. DNA was isolated from the residual liquid of whole LBC samples using the hot sodium hydroxide method [25]. Cell pellets were lysed with 50 μL of alkaline lysis solution (25 mM NaOH and 0.2 mM ethylenediaminetetraacetic acid; pH 12.0) for 10 min at 95 °C. Next, lysed cells were centrifuged at 13,200 rpm for 1 min and directly used as the DNA template. The presence of human β-globin DNA in cells was determined using PCR and served as an internal standard for genotyping. All HPV genotypes tested positive for human β-actin, demonstrating that DNA of amplifiable quality was extracted from the specimens. The HPV-typing assay method utilized uniplex E6/E7 PCR, as previously described by Okodo et al. [15]. This method can detect 39 mucosal HPV types, including 12 high-risk genotypes (i.e., HPV16, HPV18, HPV31, HPV33, HPV35, HPV39, HPV45, HPV51, HPV52, HPV56, HPV58, and HPV59) [26], from as few as 100 viral copies, with no cross-reactivity across all HPV genotypes. This PCR method may occasionally provide false-positive results; to eliminate the possibility of DNA contamination, each round of PCR was performed with negative controls using DNase-free water. Plasmid DNA containing the whole HPV genome was used as the positive control.

### 4.4. HPV Genotyping of Manually Microdissected Cytological Signs of HPV Infection

A MD-based technique was used to detect HPV directly in binucleated cells and parakeratosis on Pap smear slides [14,27]. Cell pellets were collected and analyzed from randomly selected binucleated cells and parakeratosis-positive LBC samples infected with multiple HPV genotypes, including genotypes identified by logistic regression analysis.

Each cell pellet was mounted in a single thin layer on a microscope slide and fixed with 95% ethanol. Pap smear slides were reviewed by two cytotechnologists, and certain binucleated cells and parakeratosis were selected for sampling. Selected cells were individually photographed. Thereafter, the slide was soaked in xylene to remove the cover slip. Subsequently, xylene was washed away with 100% ethanol. Thereafter, under a microscope, the chosen cells were collected by gently picking up their edge with the tip of a 27 G injection needle. The cells attached to the tip of the needle were transferred to each tube containing the alkaline lysis solution. DNA isolation and uniplex E6/E7 PCR for HPV genotyping of MD samples were performed as previously described [15].

### 4.5. Statistical Analysis

SPSS version 25.0 (IBM Corp., Armonk, NY, USA) was used for statistical analysis. Differences between groups were examined using the chi-squared test. In addition, we used stepwise logistic regression with backward elimination and a likelihood ratio test for model construction to evaluate HPV genotypes associated with various atypical cells. Goodness-of-fit was assessed using the Hosmer–Lemeshow test. All HPV genotypes were entered into the model, and statistically non-significant genotypes were removed one at a time; moreover, the OR, 95%CI, and *p*-values were estimated at each step. In all cases, a *p*-value < 0.05 indicates a statistically significant difference.

## 5. Conclusions

This study revealed that binucleated cells, parakeratinocytes, and giant cells were significantly associated with specific HPV genotypes. Nevertheless, it has been difficult to detect specific HPV genotypes by MD-PCR, and the HPV detection rate by MD-PCR was approximately 10%. These findings indicate that binucleated cells, parakeratinocytes, and giant cells may not be associated with specific HPV genotypes.

## Figures and Tables

**Table 1:** Rates of five cytological signs in Papanicolaou smears according to the cytologic classification of 651 samples.

Cytologic Classification	Binucleated Cells	Parakeratotic Cells	Giant Cells
ASC-US (*n* = 137)	82.5% (113/137)	27.7% (38/137)	53.3% (73/137)
LSIL (*n* = 202)	80.2% (162/202)	50.0% (101/202)	45.0% (91/202)
ASC-H (*n* = 54)	66.7% (36/54)	20.4% (11/54)	31.5% (17/54)
HSIL (*n* = 258)	77.5% (200/258)	31.4% (81/258)	34.5% (89/258)
Total (*n* = 651)	84.6% (511/651)	35.5% (231/651)	41.5% (270/651)

ASC-H, atypical squamous cells and high-grade squamous intraepithelial lesion could not be ruled out; ASC-US, atypical squamous cells of undetermined significance; HSIL, high-grade squamous intraepithelial lesion; LSIL, low-grade squamous intraepithelial lesion.

**Table 2:** Correlation between cytological signs of HPV infection and high-risk HPV genotypes.

		High-Risk HPV Genotypes											
		16	18	31	33	35	39	45	51	52	56	58	59
		*n* =143	*n* = 35	*n* = 53	*n* = 23	*n* = 5	*n* = 71	*n* = 6	*n* = 72	*n* = 151	*n* = 81	*n* = 143	*n* = 9
BNC	Present	79.7%	77.1%	79.2%	82.6%	80.0%	78.9%	100.0%	80.6%	84.8%	84.0%	80.4%	88.9%
		(114/143)	(27/35)	(42/53)	(19/23)	(4/5)	(56/71)	(6/6)	(58/72)	(128/151)	(68/81)	(115/143)	(8/9)
	Absent	20.3%	22.9%	20.8%	17.4%	20.0%	21.1%	0.0%	19.4%	15.2%	16.0%	19.6%	11.1%
		(29/143)	(8/35)	(11/53)	(4/23)	(1/5)	(15/71)	(0/6)	(14/72)	(23/151)	(13/81)	(28/143)	(1/9)
PC	Present	41.3%	42.9%	26.4%	39.1%	40.0%	33.8%	16.7%	30.6%	24.5%	56.8%	42.0%	33.3%
		(59/143)	(15/35)	(14/53)	(9/23)	(2/5)	(24/71)	(1/6)	(22/72)	(37/151)	(46/81)	(60/143)	(3/9)
	Absent	58.7%	57.1%	73.6%	60.9%	60.0%	66.2%	83.3%	69.4%	75.5%	43.2%	58.0%	66.7%
		(84/143)	(20/35)	(39/53)	(14/23)	(3/5)	(47/71)	(5/6)	(50/72)	(114/151)	(35/81)	(83/143)	(6/9)
GC	Present	40.6%	28.6%	45.3%	60.9%	80.0%	46.5%	50.0%	31.9%	43.0%	48.1%	42.7%	22.2%
		(58/143)	(10/35)	(24/53)	(14/23)	(4/5)	(33/71)	(3/6)	(23/72)	(65/151)	(39/81)	(61/143)	(2/9)
	Absent	59.4%	71.4%	54.7%	39.1%	20.0%	53.5%	50.0%	68.1%	57.0%	51.9%	57.3%	77.8%
		(85/143)	(25/35)	(29/53)	(9/23)	(1/5)	(38/71)	(3/6)	(49/72)	(86/151)	(42/81)	(82/143)	(7/9)

BNC, binucleated cells; GC, giant cells; HPV, human papillomavirus; PC, parakeratotic cells.

**Table 3:** Correlation between cytological signs of HPV infection and other HPV genotypes.

		**Other HPV** **Genotypes**													
		**6**	**11**	**26**	**30**	**34**	**40**	**42**	**44**	**53**	**54**	**55**	**61**	**62**	
		***n* = 22**	***n* = 4**	***n* = 0**	***n* = 14**	***n* = 21**	***n* = 14**	***n* = 58**	***n* = 10**	***n* = 59**	***n* = 21**	***n* = 25**	***n* = 30**	***n* = 44**	
BNC	Present	77.3%	75.0%	–	78.6%	76.2%	85.7%	93.1%	70.0%	76.3%	76.2%	72.0%	83.3%	81.8%	
		(17/22)	(3/4)	–	(11/14)	(16/21)	(12/14)	(54/58)	(7/10)	(45/59)	(16/21)	(18/25)	(25/30)	(36/44)	
	Absent	22.7%	25.0%	–	21.4%	23.8%	14.3%	6.9%	30.0%	23.7%	23.8%	28.0%	16.7%	18.2%	
		(5/22)	(1/4)	–	(3/14)	(5/21)	(2/14)	(4/58)	(3/10)	(14/59)	(5/21)	(7/25)	(5/30)	(8/44)	
PC	Present	45.5%	0.0%	–	28.6%	38.1%	71.4%	55.2%	70.0%	27.1%	47.6%	48.0%	36.7%	54.5%	
		(10/22)	(0/4)	–	(4/14)	(8/21)	(10/14)	(32/58)	(7/10)	(16/59)	(10/21)	(12/25)	(11/30)	(24/44)	
	Absent	54.5%	100.0%	–	71.4%	61.9%	28.6%	44.8%	30.0%	72.9%	52.4%	52.0%	63.3%	45.5%	
		(12/22)	(4/4)	–	(10/14)	(13/21)	(4/14)	(26/58)	(3/10)	(43/59)	(11/21)	(13/25)	(19/30)	(20/44)	
GC	Present	40.9%	25.0%	–	35.7%	23.8%	57.1%	39.7%	30.0%	37.3%	38.1%	52.0%	33.3%	47.7%	
		(9/22)	(1/4)	–	(5/14)	(5/21)	(8/14)	(23/58)	(3/10)	(22/59)	(8/21)	(13/25)	(10/30)	(21/44)	
	Absent	59.1%	75.0%	–	64.3%	76.2%	42.9%	60.3%	70.0%	62.7%	61.9%	48.0%	66.7%	52.3%	
		(13/22)	(3/4)	–	(9/14)	(16/21)	(6/14)	(35/58)	(7/10)	(37/59)	(13/21)	(12/25)	(20/30)	(23/44)	
		**Other HPV** **Genotypes**													
		**66**	**67**	**68**	**69**	**70**	**71**	**73**	**74**	**81**	**82**	**84**	**85**	**89**	**90**
		***n* = 22**	***n* = 15**	***n* = 33**	***n* = 2**	***n* = 10**	***n* = 24**	***n* = 9**	***n* = 51**	***n* = 24**	***n* = 39**	***n* = 17**	***n* = 0**	***n* = 17**	***n* = 37**
BNC	Present	77.3%	73.3%	75.8%	50.0%	80.0%	79.2%	77.8%	78.4%	83.3%	74.4%	82.4%	–	94.1%	73.0%
		(17/22)	(11/15)	(25/33)	(1/2)	(8/10)	(19/24)	(7/9)	(40/51)	(20/24)	(29/39)	(14/17)	–	(16/17)	(27/37)
	Absent	22.7%	26.7%	24.2%	50.0%	20.0%	20.8%	22.2%	21.6%	16.7%	25.6%	17.6%	–	5.9%	27.0%
		(5/22)	(4/15)	(8/33)	(1/2)	(2/10)	(5/24)	(2/9)	(11/51)	(4/24)	(10/39)	(3/17)	–	(1/17)	(10/37)
PC	Present	45.5%	46.7%	33.3%	50.0%	20.0%	45.8%	55.6%	47.1%	45.8%	25.6%	47.1%	–	47.1%	56.8%
		(10/22)	(7/15)	(11/33)	(1/2)	(2/10)	(11/24)	(5/9)	(24/51)	(11/24)	(10/39)	(8/17)	–	(8/17)	(21/37)
	Absent	54.5%	53.3%	66.7%	50.0%	80.0%	54.2%	44.4%	52.9%	54.2%	74.4%	52.9%	–	52.9%	43.2%
		(12/22)	(8/15)	(22/33)	(1/2)	(8/10)	(13/24)	(4/9)	(27/51)	(13/24)	(29/39)	(9/17)	–	(9/17)	(16/37)
GC	Present	40.9%	6.7%	33.3%	0.0%	40.0%	41.7%	44.4%	39.2%	54.2%	28.2%	35.3%	–	41.2%	40.5%
		(9/22)	(1/15)	(11/33)	(0/2)	(4/10)	(10/24)	(4/9)	(20/51)	(13/24)	(11/39)	(6/17)	–	(7/17)	(15/37)
	Absent	59.1%	93.3%	66.7%	100.0%	60.0%	58.3%	55.6%	60.8%	45.8%	71.8%	64.7%	–	58.8%	59.5%
		(13/22)	(14/15)	(22/33)	(2/2)	(6/10)	(14/24)	(5/9)	(31/51)	(11/24)	(28/39)	(11/17)	–	(10/17)	(22/37)

BNC, binucleated cells; HPV, human papillomavirus; GC, giant cells; PC, parakeratotic cells

**Table 4:** Odds ratios and 95% confidence intervals of HPV genotypes for each type of atypical cells in the logistic regression analysis.

Atypical Cells	WL-PCR Genotype	*p*-Value	Odds Ratio (95%CI)
Binucleated cells	HPV52	0.002	2.01 (1.28–3.15)
	HPV11	0.049	0.14 (0.02–1.00)
	HPV42	0.017	2.69 (1.19–6.07)
Parakeratotic cells	HPV16	0.010	1.75 (1.14–2.68)
	HPV56	0.000	3.26 (1.92–5.53)
	HPV58	0.028	1.63 (1.05–2.51)
	HPV40	0.011	5.19 (1.45–18.59)
	HPV42	0.018	2.09 (1.13–3.85)
	HPV62	0.017	2.30 (1.16–4.53)

95%CI, 95% confidence interval; HPV, human papillomavirus; PCR, polymerase chain reaction; WL, whole liquid-based cytology

**Table 5:** Detection of binucleated cells in multiple HPV infections using MD-PCR.

Case Number	Cytologic Classification	HPV Genotype	
		WL-PCR	MD-PCR (MD-PCR Positive/MD Samples)
1	HSIL	52, 82	−(0/15)
2	ASC-H	51, 52, 84	51 (4/7)
3	ASC-US	52, 58	−(0/6)
4	ASC-US	39, 52, 62	−(0/2)
5	HSIL	52, 42	−(0/2)
6	LSIL	58, 6, 42, 68	6 (2/8)
7	HSIL	39, 56, 58, 42	−(0/3)
8	LSIL	16, 6, 42, 54, 61	−(0/6)
9	LSIL	51, 42, 62	−(0/8)

Numbers in parentheses indicate the ratio of the number of MD samples to the number of positive MD-PCR results in each case. ASCH, atypical squamous cells and high-grade squamous intraepithelial lesion could not be ruled out; ASCUS, atypical squamous cells of undetermined significance; HPV, human papillomavirus; HSIL, high-grade squamous intraepithelial lesion; LSIL, low-grade squamous intraepithelial lesion; MD, microdissection; PCR, polymerase chain reaction; WL, whole liquid-based cytology.

**Table 6:** Detection of parakeratotic cells in multiple HPV infections using MD-PCR.

Case Number	Cytologic Classification	HPV Genotype	
		WL-PCR	MD-PCR (MD-PCR Positive/MD Samples)
1	LSIL	31, 62, 71	31 (1/5)
2	HSIL	39, 56, 58, 42	−(0/3)
3	ASC-US	52, 58, 42, 44, 55, 62, 71, 74	−(0/6)
4	ASC-US	56, 62, 81	56 (1/10)
5	LSIL	18, 56	56 (1/4)
6	HSIL	39, 56, 58, 42	−(0/18)
7	HSIL	16, 39	−(0/3)
8	HSIL	58, 74	−(0/4)
9	HSIL	31, 56	−(0/1)
10	HSIL	58, 42, 68	−(0/28)
11	LSIL	42, 74, 81	−(0/6)
12	HSIL	51, 58, 42	−(0/3)
13	LSIL	34, 42, 53, 67	−(0/3)
14	LSIL	39, 52, 58, 55, 74	52 (7/28), 74 (3/28)
15	LSIL	33, 39, 59, 40, 42, 68, 73, 81	−(0/11)
16	LSIL	16, 52, 40, 54, 70	−(0/30)
17	LSIL	52, 58, 40, 54, 90	−(0/7)
18	HSIL	16, 59,	16 (1/19)
19	LSIL	16, 42	42 (4/41)
20	HSIL	16, 82	16 (1/17)

Numbers in parentheses indicate the ratio of the number of MD samples to the number of positive MD-PCR results in each case. ASCUS, atypical squamous cells of undetermined significance; HPV, human papillomavirus; HSIL, high-grade squamous intraepithelial lesion; LSIL, low-grade squamous intraepithelial lesion; MD, microdissection; PCR, polymerase chain reaction; WL, whole liquid-based cytology.

## Data Availability

The data presented in this study are available on request from the corresponding author.
